# A Comparison of 7 Tesla MR Spectroscopic Imaging and 3 Tesla MR Fingerprinting for Tumor Localization in Glioma Patients

**DOI:** 10.3390/cancers16050943

**Published:** 2024-02-26

**Authors:** Philipp Lazen, Pedro Lima Cardoso, Sukrit Sharma, Cornelius Cadrien, Thomas Roetzer-Pejrimovsky, Julia Furtner, Bernhard Strasser, Lukas Hingerl, Alexandra Lipka, Matthias Preusser, Wolfgang Marik, Wolfgang Bogner, Georg Widhalm, Karl Rössler, Siegfried Trattnig, Gilbert Hangel

**Affiliations:** 1 High-Field MR Center, Department of Biomedical Imaging and Image-Guided Therapy, Medical University of Vienna, 1090 Vienna, Austria; philipp.lazen@meduniwien.ac.at (P.L.);; 2 Department for Neurosurgery, Medical University of Vienna, 1090 Vienna, Austria; 3 Christian Doppler Laboratory for MR Imaging Biomarkers, 1090 Vienna, Austria; 4 Division of Neuropathology and Neurochemistry, Department of Neurology, Medical University of Vienna, 1090 Vienna, Austria; 5 Division of Neuroradiology and Musculoskeletal Radiology, Department of Biomedical Imaging and Image-Guided Therapy, Medical University of Vienna, 1090 Vienna, Austria; 6 Research Center for Medical Image Analysis and Artificial Intelligence (MIAAI), Danube Private University, 3500 Krems, Austria; 7 Division of Oncology, Department of Internal Medicine I, Medical University of Vienna, 1090 Vienna, Austria; 8 Institute for Clinical Molecular MRI, Karl Landsteiner Society, 3100 St. Pölten, Austria

**Keywords:** magnetic resonance imaging, magnetic resonance spectroscopy, chemical shift imaging, glioma, magnetic resonance fingerprinting

## Abstract

**Simple Summary:**

This study compared two brain imaging methods, 7T magnetic resonance spectroscopic imaging (MRSI), which can image metabolic processes, and 3T magnetic resonance fingerprinting (MRF), which can image magnetic relaxation times, in 12 people with brain tumors called gliomas. Our goal was to understand how well these two approaches corresponded to each other, and which metabolite or relaxation time map was closest to the clinical standard, a neuroradiologist’s tumor segmentation. In order to do this, we defined hotspots for each method and compared their overlaps. Additionally, we investigated the region around the tumor to look for evidence of possible tumor infiltration. The results of this study could improve how we use magnetic resonance imaging to monitor gliomas in patients.

**Abstract:**

This paper investigated the correlation between magnetic resonance spectroscopic imaging (MRSI) and magnetic resonance fingerprinting (MRF) in glioma patients by comparing neuro-oncological markers obtained from MRSI to T1/T2 maps from MRF. Data from 12 consenting patients with gliomas were analyzed by defining hotspots for T1, T2, and various metabolic ratios, and comparing them using Sørensen–Dice similarity coefficients (DSCs) and the distances between their centers of intensity (COIDs). The median DSCs between MRF and the tumor segmentation were 0.73 (T1) and 0.79 (T2). The DSCs between MRSI and MRF were the highest for Gln/tNAA (T1: 0.75, T2: 0.80, tumor: 0.78), followed by Gly/tNAA (T1: 0.57, T2: 0.62, tumor: 0.54) and tCho/tNAA (T1: 0.61, T2: 0.58, tumor: 0.45). The median values in the tumor hotspot were T1 = 1724 ms, T2 = 86 ms, Gln/tNAA = 0.61, Gly/tNAA = 0.28, Ins/tNAA = 1.15, and tCho/tNAA = 0.48, and, in the peritumoral region, were T1 = 1756 ms, T2 = 102 ms, Gln/tNAA = 0.38, Gly/tNAA = 0.20, Ins/tNAA = 1.06, and tCho/tNAA = 0.38, and, in the NAWM, were T1 = 950 ms, T2 = 43 ms, Gln/tNAA = 0.16, Gly/tNAA = 0.07, Ins/tNAA = 0.54, and tCho/tNAA = 0.20. The results of this study constitute the first comparison of 7T MRSI and 3T MRF, showing a good correspondence between these methods.

## 1. Introduction

Over the last few decades, different approaches to magnetic resonance imaging (MRI), a non-invasive diagnostic technique that uses a strong magnetic field, dynamic gradient fields, and radio frequency pulses to create detailed images of the body’s internal structures with different types of contrasts, have been developed. Amongst other applications, MRI is a vital tool in the diagnosis, grading, and treatment monitoring of glioma, a type of brain tumor [1,2,3,4]. Modern MRI approaches include 3T MR fingerprinting (MRF) and 7T high-resolution MR spectroscopic imaging (MRSI), which aim to accumulate more specific information about gliomas than conventional T1/T2-weighted MR imaging, enhancing our understanding of these conditions [5,6]. 

### 1.1. Magnetic Resonance Spectroscopic Imaging

MRSI provides metabolic information beyond contrast-enhanced T1/T2 MRI. The methodology visualizes different neurochemical concentrations without the need for contrast agents, and is thus a powerful tool in the investigation of diseases that influence metabolite and neurotransmitter distributions in the brain, such as gliomas. Notably, certain metabolites, such as N-acetylaspartate (NAA), creatine (Cr), choline (Cho), glutamine (Gln), glycine (Gly), and myo-inositol (Ins), are well suited as neuro-oncological markers due to the differences in their concentrations between tumor and healthy brain tissue and because of their stability in spectroscopic imaging, based on the accumulated experience of 7T MRSI in gliomas [6,7].

Glutamine and glycine are amino acids that are involved in many metabolic processes in cells, including protein synthesis, energy production, and cell growth and re-pair [8,9]. For cancer cells, both glutamine and glycine can be their primary source of energy, and these compounds also play a role in the proliferation of cancer cells [10]. Choline, on the other hand, is a polyatomic ion that plays an important role as a precursor of the phospholipid phosphatidylcholine, a major component of cell membranes, which is vital for their structural integrity and fluidity. Cancer cells tend to have a high demand for choline to sustain their proliferation [11]. Ins is an abundant metabolite in the brain and has various biochemical functions including signal transduction, protein phosphorylation, gene expression, chromatin remodeling, and mRNA transport. It is found mostly in astrocytes and increased levels have been related to reactive gliosis and brain tumors, as well as neurodegenerative diseases and multiple sclerosis [12,13]. N-acetylaspartate (NAA) is used in magnetic resonance spectroscopy as a biomarker of neuronal health, integrity, and viability [14]. Using ratios between these metabolites instead of their absolute concentrations is more common in routine clinical practice due to their simpler acquisition and processing [15].

The MRSI approach we used acquires free induction decay (FID) signals, following concentric ring trajectories (CRTs) in k-space [16]. Apart from the method’s high sensitivity, one of its main advantages is its time efficiency. CRT-FID-MRSI can achieve high-resolution metabolic maps with a 64 × 64 × 39 matrix that covers the whole brain using an isotropic voxel size of 3.4 mm in 15 min, which presents a significant improvement compared to clinically available MRSI approaches. Due to the increased signal-to-noise ratio (SNR) and spectral resolution at higher field strengths, MRSI benefits from the use of modern ultra-high-field 7T systems. For example, it is possible to separate glutamate (Glu) and Gln at 7T, which is difficult at 3T due to the spectral overlap of the resonances of these metabolites [17].

### 1.2. Magnetic Resonance Fingerprinting

MRF, on the other hand, is a modern approach to mapping magnetic tissue properties, such as the T1 and T2 relaxation times [5]. Unlike conventional T1 and T2 mapping sequences, MRF derives the parameters of interest from a single acquisition wherein the flip angle, the repetition time (TR), and the echo time (TE) are varied pseudo-randomly during the acquisition of heavily undersampled data. The resulting data “fingerprint” can then be compared to a database, yielding T1 and T2 values. Since the result of this procedure is an actual T1 or T2 map and not just a T1- or T2-weighted image, MRF is considered a quantitative methodology, as it quantitatively estimates real physical quantities rather than providing arbitrary intensity parameters. These quantitative estimates are more useful as a basis for machine-learning models. Similar to CRT-MRSI, MRF uses non-Cartesian k-space sampling to improve upon conventional T1 and T2 mapping sequences by minimizing the total acquisition duration. 

Morphologically, the T1 and T2 relaxation times can change as a result of a change in the microenvironment of the tumor. For example, an accumulation of water in the cancer increases both the T1 and T2 times, as relaxation times in free water are longer than in bound water [18,19].

### 1.3. Motivation and Purpose

Previously, the results obtained from MRSI acquisitions were compared to those of clinical positron emission tomography (PET) scans [7,20]. Now, we aimed to investigate the correlation between MRSI and MRF in glioma patients in this study, focusing on the correspondence between the hotspots identified in both methods. This constitutes an initial comparison of 7T MRSI and 3T MRF to determine whether these methods complement each other or whether they correlate.

The purpose of this work was to investigate, for the first time, whether metabolic changes detected by 7T MRSI correspond to structural changes found by 3T MRF in glioma patients by correlating the metabolic ratios of MRSI to T1 and T2 maps of MRF.

## 2. Materials and Methods

### 2.1. Study Population

We acquired the approval of the institutional review board of the Medical University of Vienna (protocol 1991/2018), as well as written, informed consent from all participants of this prospective study. Participants were selected consecutively between February and December 2019. The inclusion criteria were a suspected glioma diagnosis, as well as informed consent, and the absence of MRI contraindications. Subjects were excluded if they were not eligible for a 7T MRI, if the MRSI data quality was too poor to allow a reasonable data analysis based on the rejection criteria explained in Section 2.2, or if the subject’s tumor could not be histologically confirmed as a glioma.

**Figure 1:**
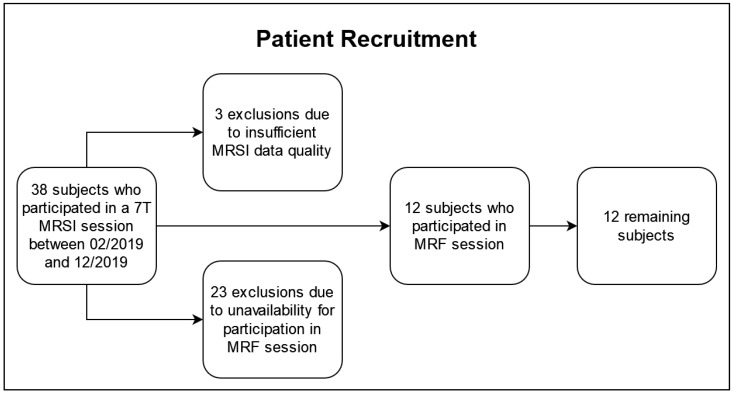
Flowchart of the recruitment for this study.

The patient recruitment is illustrated in Figure 1. Of the 38 subjects who underwent a 7T MRSI protocol, three were excluded based on our quality criteria described in Section 2.2 and 23 were unavailable for the additional MRF session. The remaining 12 subjects (five females, seven males), 48 ± 15 years of age, participated in a 3T and a 7T session within 50 h (median: same day) to guarantee consistency and comparability across the data sets. In the cohort, there were two IDH-mutant grade 2 astrocytomas, three IDH-mutant grade 3 astrocytomas, two IDH-mutant grade 2 oligodendrogliomas, one IDH-mutant grade 3 oligodendroglioma, and four IDH-wildtype grade 4 glioblastomas, according to the 2021 WHO classification of gliomas [21]. The patient cohort is listed in Table 1, and it overlapped with a cohort in previous papers (see Table S1) [6,7]. 

### 2.2. MRSI Protocol and Data Processing 

The MRSI protocol was performed on a 7T Magnetom scanner (Siemens Healthineers, Erlangen, Germany) using a 1 Tx/32 Rx head coil (Nova Medical, Wilmington, MA, USA) and consisted of a T1-weighted MP2RAGE as the morphological reference, a B0 field map, a B1 field map for flip-angle optimization, and a CRT-FID-MRSI scan (TR = 450 ms, acquisition delay AD = 1.3 ms; FOV = 220 × 220 × 133 mm^3^, resolution = 3.4 × 3.4 × 3.4 mm^3^, TA = 15 min) [6,16]. 

MRSI post-processing used the previously introduced in-house pipeline and involved quantification in the spectral range of 1.8–4.1 ppm using LC Model [22]. A metabolite basis set consisting of 17 metabolites and a measured macromolecular baseline was used for fitting [23,24]. The metabolites included the previously mentioned neuro-oncological markers Cho (glycerol–phosphocholine and phosphocholine, summarized as total choline, tCho), Cr (creatine and phosphocreatine, summarized as total creatine, tCr), Gln, Gly, Ins, and NAA (NAA together with NAA–glutamate, summarized as total NAA, tNAA), as well as γ-aminobutyric acid, glutathione, scyllo-inositol, serine, taurine, 2-hydroxyglutarate, and glutamate. An overview of the processing parameters is given in Table S2 [25]. 

The spectral range selected for quantification is situated between the resonance frequencies of water and lipids. An expansion of this spectral window upfield to encompass the lactate resonance at 1.3 ppm would be problematic due to the introduction of artifacts attributable to lipid signals and necessitate the implementation of advanced signal processing techniques, such as L2 regularization. Conversely, extending the spectral range downfield would increase the risk of signal contamination from the residual water peak. For each metabolite map, voxels were discarded if their tCr FWHM was <0.15 ppm, their tCr SNR was <5, the metabolite CRLBs were above 80%, or the metabolite fit coefficient was >13 median absolute standard deviations [6]. Lastly, the metabolic maps were qualitatively assessed, and the patient’s dataset was discarded if the proportion of excluded voxels prevented a reasonable analysis, which may happen due to motion, lipid contamination, or B_0_ inhomogeneities. 

Data analysis included the ratios of tCho/tNAA, Gln/tNAA, Gly/tNAA, Ins/tNAA, tCho/tCr, Gln/tCr, Gly/tCr, and Ins/tCr, as they are commonly used [2,11,12]. We specifically focused on the metabolite ratios to NAA because a drop in NAA, which corresponds to neuronal loss and is commonly seen in tumors, synergizes well with increases in tCho, Gln, Gly, and Ins, often producing well-defined hotspots in the ratio maps.

### 2.3. MRF and Clinical Protocol

The MRF scan was performed on a 3T Magnetom PrismaFit MR scanner using a 1 Tx/64 Rx head coil (Siemens Healthineers, Erlangen, Germany), and was based on a 2D Fast Imaging with Steady-state Precession (FISP) spiral readout (FOV = 256 × 256 mm, in-plane resolution = 1 × 1 mm, TA = 20 s per slice). To reduce the MRF’s long acquisition duration to an acceptable time, the number of acquired slices was kept as low as possible while still covering the entire tumor.

In addition to the MRF and MRSI protocol, a routine clinical 3T MRI was performed, consisting of a native T1-weighted image, a contrast-enhanced T1-weighted image, and a fluid-suppressed T2-weighted image. The clinical images were segmented by a neuroradiologist. Co-registered clinical morphological scans and segmentations were used to define the following regions of interest (ROIs): tumor segmentation (“TU”), which included contrast-enhancing and non-contrast-enhancing tissue within the tumor, as well as necrosis and edema; dilated tumor segmentation (“TU + PT”), which added the peritumoral region by first dilating TU by six voxels (effectively adding an approximately 2 cm thick layer surrounding the tumor); and peritumoral segmentation alone (“PT”), which we created by removing the original from the dilated tumor segmentation. 

### 2.4. Data Analysis

We compared these segmentations to a normal-appearing white matter (NAWM) reference region, which was created by subtracting TU + PT from a white matter mask and eroding the resulting region once. Additionally, we investigated the metabolic abnormalities given by the median metabolite ratios and relaxation times in the different ROIs. Within each segmentation, we defined hotspots by including all voxels with a value greater than 150% of the respective median value of the NAWM reference region. For analysis, we compared the resulting T1 and T2 hotspots with the metabolite hotspots and with the tumor segmentation using Sørensen–Dice similarity coefficients (DSC), analogous to a previously established approach [7,26,27].

$$DSC=\frac{2\times \left|N_{MRSI}\cap N_{MRF}\right|}{\left|N_{MRSI}\right|+\left|N_{MRF}\right|}$$

Since DSCs measure only the overlap of two regions, we also calculated the centers of intensity (i.e., the average position of all points of the ROI) of each region, according to

$${\vec{r}}_{VOI}=\frac{\sum_{i\;\in \;VOI}{\vec{v}}_{i}\times I\left({\vec{v}}_{i}\right)}{\sum_{i\;\in \;VOI}I\left({\vec{v}}_{i}\right)},$$

with the voxel vectors ${\vec{v}}_{i}$ and the intensities $I\left({\vec{v}}_{i}\right)$, and then evaluated their distances from each other (“center of intensity distances”, COIDs):

$$COID=\left|{\vec{r}}_{MRSI}-{\vec{r}}_{MRF}\right|$$

Due to the possible tumor infiltrations of the surrounding regions, we extended our analysis to the peritumoral regions, again looking at DSCs between the MRF’s T1 and T2 hotspots and the MRSI’s metabolic hotspots. In addition to the similarity measures, we evaluated the median relaxation times and metabolic ratios in the hotspots within the different regions of interest (TU, TU + PT, PT) and the NAWM reference region. Last, we compared TU and PT using a two-sided paired Student’s *t*-test. Since our approach of using a threshold to define the hotspots in TU and PT naturally increased the median values in these regions compared to the un-thresholded regions, the comparison to NAWM would have been meaningless and was thus omitted. 

## 3. Results

**Figure 2:**
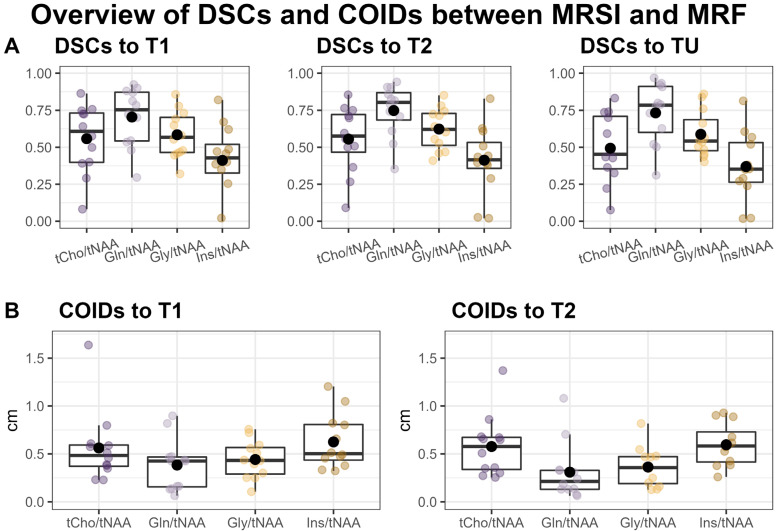
Overview of Sørensen–Dice similarity coefficients (DSCs) between the MRSI’s metabolite ratio hotspots, MRF’s T1 and T2 hotspots, and the tumor segmentation (TU) (**A**) and the distances of the centers of intensity (COIDs) between the MRSI and MRF hotspots (**B**).

Overall, we found a very high correspondence between the hotspots in the ratio maps for both Gln/tNAA and Gly/tNAA and the MRF’s T1 and T2 maps, as well as the tumor segmentation, which is reflected in the respective DSCs and COIDs (see Figure 2 and Table 2). 

### 3.1. Median Relaxation Times and Metabolic Ratios

Regarding the metabolic ratio values, the cohort’s median in the tumor hotspot was the highest for Ins/tNAA (median = 1.15, [Q1, Q3] = [1.04, 1.21]), followed by Gln/tNAA (0.61, [0.56, 0.70]), tCho/tNAA (0.48, [0.42, 0.55]), and Gly/tNAA (0.28, [0.20, 0.36]), and the respective relaxation times were T1 = 1724 ms (Q1 = 1690 ms, Q3 = 1804 ms) and T2 = 85 ms (Q1 = 80 ms, Q3 = 106 ms). The corresponding values in NAWM were 0.54, [0.51, 0.59] for Ins/tNAA; 0.16, [0.13, 0.20] for Gln/tNAA; 0.20, [0.18, 0.21] for tCho/tNAA; and 0.07, [0.06, 0.10] for Gly/tNAA; and the relaxation times were T1 = 950 ms (Q1 = 941 ms, Q3 = 972 ms) and T2 = 42.9 ms (Q1 = 42.6 ms, Q3 = 43.3 ms). For an overview of these numbers, see Table 3. 

**Figure 3:**
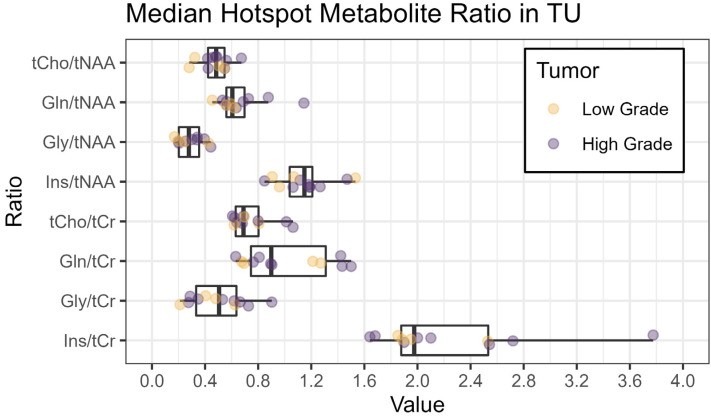
Boxplots of median metabolite ratios for tCho/tNAA, Gln/tNAA, Gly/tNAA, and Ins/tNAA, as well as tCho/tCr, Gln/tCr, Gly/tCr, and Ins/tCr, within the hotspot in the tumor segmentation TU. The colors indicate the tumor grade (yellow: low grade, grade 2 or below; violet: high grade, grade 3 or above).

**Figure 4:**
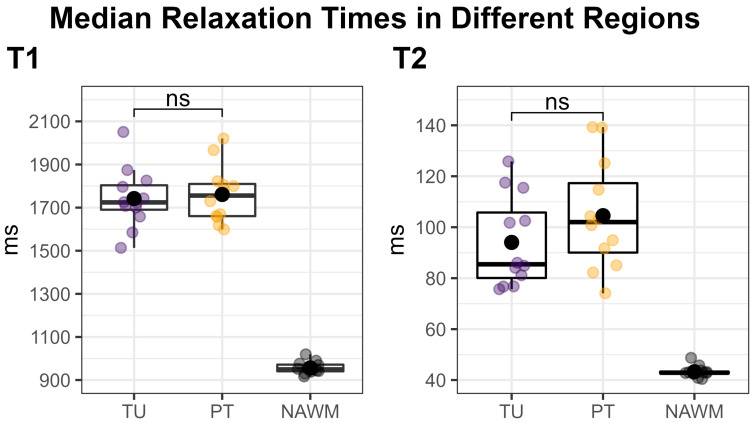
Median T1 (**left**) and T2 (**right**) relaxation times within the tumor (TU, violet) and peritumoral (PT, yellow) segmentations’ hotspots, compared to the normal-appearing white matter (NAWM) control region (black). Each dot corresponds to one patient. TU and PT were compared using a two-sided paired t-test, which showed no significant difference (“ns”). Our approach of using a threshold to define the hotspots in TU and PT naturally increased the median values in these regions compared to the un-thresholded regions, which would have rendered a comparison to NAWM meaningless.

**Figure 5:**
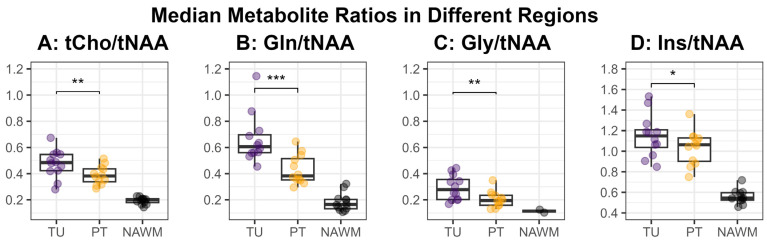
Median values for the metabolite ratios tCho/tNAA (**A**), Gln/tNAA (**B**), Gly/tNAA (**C**), and Ins/tNAA (**D**) within the defined hotspots in the tumor (TU, violet) and peritumoral regions (PT, yellow), as well as the normal-appearing white matter control region (NAWM, black). The medians in the regions TU and PT were compared using a two-sided paired *t*-test and the resulting significance levels were noted in the plot (*: *p* < 0.05, **: *p* < 0.01, ***: *p* < 0.001).

Figure 3 shows an overview of the medians of the metabolite ratios in the tumor hotspot while illustrating the different tumor grades by color-coding. The cohort’s median metabolite ratios and median relaxation times for the hotspots in the TU, the PT, and the NAWM are noted in Table 3 and shown in more detail in Figure 4 and Figure 5. Notably, we found statistically significant differences between TU and PT in the metabolite ratios (with the values in TU higher than in PT), but no such effect was found for the relaxation times.

### 3.2. Similarity Measures

When comparing the hotspots of MRSI within the tumor to the entire segmentation TU, we found the highest DSC for Gln/tNAA (median = 0.78, [Q1, Q3] = [0.60, 0.91]), followed by Gly/tNAA (0.54, [0.48, 0.69]), tCho/tNAA (0.45, [0.35, 0.71]), and Ins/tNAA (0.35, [0.26, 0.53]). The DSCs for MRF were similar for both T1 (0.73, [0.66, 0.83]) and T2 (0.79, [0.67, 0.86]). 

Comparing MRSI to the MRF’s T1 hotspots in the tumor yielded the highest DSCs for Gln/tNAA (0.75, [0.54, 0.87]) and tCho/tNAA (0.61, [0.40, 0.73]), followed by Gly/tNAA (0.57, [0.46, 0.70]) and Ins/tNAA (0.43, [0.33, 0.52]). For T2, the DSCs were highest for Gln/tNAA (0.80, [0.68, 0.87]) and Gly/tNAA (0.62, [0.51, 0.73]), followed by tCho/tNAA (0.58, [0.47, 0.72]) and Ins/tNAA (0.41, [0.36, 0.53]). These results, together with the analogous results for the PT region, are noted in Table 2, and barplots of the entire cohort’s tumor DSCs are displayed in Figure S1. 

The centers of intensity compared to the T1 hotspot were closest for Gln/tNAA (COIDS: median = 0.43 cm, [Q1, Q2] = [0.16 cm, 0.47 cm]) and Gly/tNAA (0.43, [0.29, 0.57]), and a bit higher for tCho/tNAA (0.48, [0.37, 0.59]) and Ins/tNAA (0.50, [0.44, 0.81]). For the T2 hotspot, the lowest COIDs were found for Gln/tNAA (0.21, [0.13, 0.33]) and Gly/tNAA (0.36, [0.19, 0.47]), and the values were again higher for tCho/tNAA (0.58, [0.34, 0.67]) and Ins/tNAA (0.58, [0.42, 0.73]). These values are illustrated in Figure 2. 

### 3.3. Complementary Information

**Figure 6:**
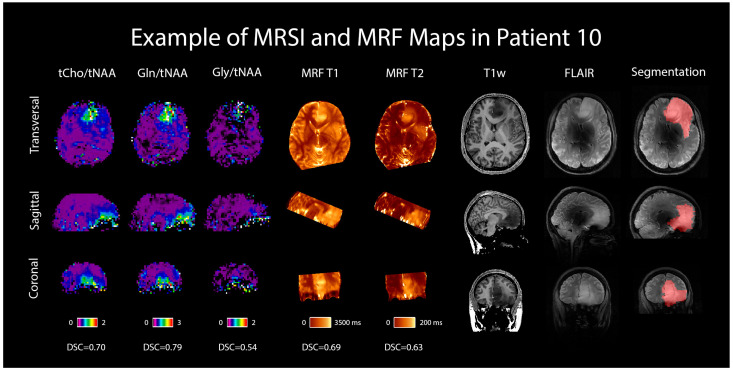
MRSI and MRF maps in a 28-year-old female patient with a histologically confirmed grade 3 astrocytoma. For comparison, 7T T1w MP2RAGE and FLAIR images are shown, as well as the radiologist’s segmentation. Transversal, sagittal, and coronal views are shown, and Sørensen–Dice similarity coefficients comparing the hotspots to the segmentation are listed below the respective maps.

An example case is shown in Figure 6 in the form of the dataset of one selected patient with an IDH-mutant grade 3 astrocytoma, including the metabolic ratio maps of tCho/tNAA, Gln/tNAA, and Gly/tNAA, T1 and T2 maps from MRF, a T1w MP2RAGE, a T2w FLAIR (both acquired at 7T), and the radiologist’s segmentation. 

Last, Figure S2 shows the median metabolic ratios for different ROIs for the threshold of 1.50 and illustrates the influence of the hotspot threshold on the median hotspot values. Part A of this figure notably shows the metabolites that exhibit the largest differences between TU and PT, and part B illustrates the case of a threshold value of 0.00, which gives an indication of what the median values in the entire TU and PT regions (rather than the hotspot) would be.

## 4. Discussion

We successfully conducted the first comparison of 7T MRSI and 3T MRF in 12 glioma patients and found a high correspondence between the metabolic hotspots of Gln/tNAA and Gly/tNAA, the T1 and T2 relaxation time hotspots, and the radiologist’s tumor segmentation, resulting in high DSCs and low COIDs for those two metabolite ratios, as shown in Table 2 and Figure 2. This finding complements previous work [7], which showed a better correspondence of Gln/tNAA and Gly/tNAA to amino acid PET than the clinically used tumor marker tCho/tNAA. In Figure 3, we provide an overview of the median hotspot metabolite ratios in low and high-grade gliomas, and we also reported median values for T1 and T2 (Table 3 and Figure 4) and for metabolite ratios (Table 3 and Figure 5). 

Glutamine and glycine are amino acids that are involved in many metabolic processes in cells, including protein synthesis, energy production, and cell growth and repair [8,9]. For cancer cells, both glutamine and glycine can be the primary source of energy, and they also play a role in the proliferation of cancer cells [10]. Choline, on the other hand, is a polyatomic ion that plays an important role as a precursor of the phospholipid phosphatidylcholine, a major component of cell membranes, which is vital for their structural integrity and fluidity. Cancer cells tend to have a high demand for choline to sustain their proliferation [11].

Our analysis of both MRF and MRSI data in the TU and PT segmentations showed similar T1 and T2 values but significantly different metabolic ratios in the hotspots of both regions (Figure 3 and Figure 4). The median values for the metabolic ratios and relaxation times were much higher in these hotspots than in the NAWM control region due to the use of thresholding for hotspot definition. 

Unfortunately, the existing literature on MRF in gliomas is still very limited [28]. De Blank et al. conducted MRF scans in a cohort of children and young adults with mostly low-grade gliomas and found that T1 and T2 values tended to increase in tumors compared to a white matter control region. While there were some differences between their median values and ours in the tumor (T1: 1444 ± 254 ms, T2: 61 ± 22 ms), the values in NAWM are comparable to those found in this study [29]. Springer et al. also found that T1 and T2 values from MRF increased in tumors compared to NAWM [30], and Marik et al. showed that MRF is feasible in differentiating between low and high-grade gliomas with an accuracy of 82% [31]. Regarding MRSI, the median values we found in this study are in accordance with previous findings that Gln/tNAA and Gly/tNAA hotspots correspond well to PET in gliomas [7]. Due to the significant overlap of the cohorts of these studies, this primarily indicates the consistency of our data evaluation and processing when compared to our 2020 paper, but to further support our hypothesis that these metabolites may be useful biomarkers, further studies including larger cohorts are required [6]. In addition, increases in tCho/tNAA have been commonly reported in the literature [32,33].

Both modalities, MRSI and MRF, are quantitative methods in contrast to the qualitative assessment of gliomas by conventional MR imaging protocols. The good correspondence between MRSI and MRF allows the application of MRF with T1 and T2 mapping at a clinically widespread available field strength of 3T, whereas high-resolution MRSI is still restricted to 7T. Another benefit of MRF for clinical use is the relatively short examination time, which requires only one sequence and provides T1 and T2 relaxation time values with superior spatial resolution. Thus, this technique can be easily incorporated into a routine brain tumor protocol, providing additional ultrastructural information in gliomas. Additionally, new advances in radiomics using texture analysis methods such as the Grey Level Co-occurrence Matrix can further improve the sensitivity and specificity of MRF in the work-up of gliomas, not only for primary differential diagnosis but also in providing additional information for monitoring over time [34].

### Limitations and Outlook

Due to the various types and grades of gliomas in this study, as well as the small cohort size, it was not possible to separately analyze each tumor’s diagnosis or grade. Our findings will need to be validated by a larger cohort to enable us to draw generalized conclusions, but we view this exploratory study as a first step toward such validation. Due to the thresholding approach for hotspot definition, p-values could be calculated only to compare TU and PT, but not for the control region. Additionally, we relied on data from NAWM for the hotspot definition, which has high CRLBs in the case of metabolites that are generally not detectable in the healthy brain, like Gln and Gly. We investigated the robustness of their fits in the supplementary material of [6], and expect an underestimation of 10–20% for Gln and Gly considering the SNR and CRLB we encounter in NAWM. We expect this to affect both the tumor and NAWM similarly, causing this effect to mostly cancel out with regard to the hotspots’ sizes and positions. 

Furthermore, MRSI is still an experimental modality and the quality of the results can vary between subjects from very good to unacceptably bad, necessitating the exclusion of some data sets. In addition, the availability of (clinical) 7T MRI systems is still limited, which, together with the rather long measurement times for MRF and MRSI, reduces the clinical applicability of this research for the time being. Last, the commonly used metabolic ratios present a weakness insofar as the overall ratio significantly depends on its denominator. In the case of the NAA ratios used in this study, all hotspots significantly depend on the coldspots in the NAA maps, introducing a correlation between them. Additionally, in voxels without an NAA fit (e.g., in the presence of lipid artifacts), no ratio can be calculated. Compared to ratios, concentration estimates (CEs) offer more reliability and should be explored in future work [35]. Unfortunately, they come with their own challenges; in the case of internal water referencing, one needs to derive a water concentration in tumor tissue, which can be difficult in practice [36,37,38].

## 5. Conclusions 

This preliminary study will provide a starting point for further studies, aiding in the development of more specific hypotheses that may be tested in larger cohort studies in the future, which should hopefully lead to better MRI-based delineation and classification of brain tumors. Ultimately, this work reinforces previous findings that glutamine and glycine show great promise as potential biomarkers in glioma imaging via the use of ultra-high-field MR spectroscopy.

## Figures and Tables

**Table 1:** An overview of the cohort containing 12 patients, including the histological diagnosis according to the WHO 2021 classification, the tumor grade, the IDH1 mutation status (IDH1 mutant, Mut; or wild type, WT), the age at the time of the 7T MRSI measurement in years (average: 48 ± 15), and the patient’s sex (5 females, F; and 7 males, M).

Cohort Overview					
Patient ID	Classification	Grade	IDH	Age	Sex
1	Glioblastoma	4	WT	47	F
2	Anaplastic astrocytoma	3	Mut	46	F
3	Anaplastic astrocytoma	3	Mut	29	M
4	Glioblastoma	4	WT	52	M
5	Diffuse astrocytoma	2	Mut	33	M
6	Glioblastoma	4	WT	58	M
7	Diffuse astrocytoma	2	Mut	77	F
8	Oligodendroglioma	3	Mut	51	M
9	Glioblastoma	4	WT	61	M
10	Anaplastic astrocytoma	3	Mut	28	F
11	Oligodendroglioma	2	Mut	38	F
12	Oligodendroglioma	2	Mut	61	M

**Table 2:** Sørensen–Dice similarity coefficients (DSCs, median and interquartile range IQR) of relaxation time hotspots (T1, T2), metabolite ratio hotspots (tCho/tNAA, Gln/tNAA, Gly/tNAA, Ins/tNAA), and different regions of interest (ROI), namely the tumor segmentation TU (containing non contrast-enhancing, contrast-enhancing, and necrotic tissue), the peritumoral region PT, and the combined region (TU + PT).

DSCs between Different Hotspots			
Segmentations	TU	TU + PT	PT
DSC between	Median (Q1, Q3)	Median (Q1, Q3)	Median (Q1, Q3)
T1 and ROI	0.73 (0.66, 0.83)	0.47 (0.44, 0.52)	0.58 (0.45, 0.65)
T2 and ROI	0.79 (0.67, 0.86)	0.46 (0.42, 0.54)	0.58 (0.43, 0.62)
tCho/tNAA and ROI	0.45 (0.35, 0.71)	0.24 (0.16, 0.33)	0.28 (0.13, 0.35)
Gln/tNAA and ROI	0.78 (0.60, 0.91)	0.55 (0.38, 0.59)	0.65 (0.46, 0.80)
Gly/tNAA and ROI	0.54 (0.48, 0.69)	0.33 (0.28, 0.38)	0.41 (0.34, 0.44)
Ins/tNAA and ROI	0.35 (0.26, 0.53)	0.21 (0.12, 0.23)	0.25 (0.10, 0.28)
tCho/tNAA and T1	0.61 (0.40, 0.73)	0.39 (0.25, 0.47)	0.29 (0.13, 0.36)
Gln/tNAA and T1	0.75 (0.54, 0.87)	0.60 (0.54, 0.64)	0.51 (0.41, 0.56)
Gly/tNAA and T1	0.57 (0.46, 0.70)	0.45 (0.38, 0.49)	0.35 (0.31, 0.39)
Ins/tNAA and T1	0.43 (0.33, 0.52)	0.32 (0.15, 0.37)	0.25 (0.10, 0.30)
tCho/tNAA and T2	0.58 (0.47, 0.72)	0.39 (0.26, 0.47)	0.28 (0.14, 0.33)
Gln/tNAA and T2	0.80 (0.68, 0.87)	0.61 (0.46, 0.64)	0.47 (0.34, 0.56)
Gly/tNAA and T2	0.62 (0.51, 0.73)	0.45 (0.39, 0.51)	0.34 (0.29, 0.39)
Ins/tNAA and T2	0.41 (0.36, 0.53)	0.33 (0.17, 0.38)	0.25 (0.12, 0.29)

**Table 3:** Median values and first and third quartile (Q1, Q3) for the cohort’s median T1 and T2 relaxation times and metabolic ratios in the hotspots within the tumor (TU), the peritumoral region (PT), and the p-values when comparing TU and PT using a two-sided paired Student’s *t*-test, as well as the median values in the hotspots in the combined TU + PT region and in the normal appearing white matter control region (NAWM). *p*-values below 0.01 are in bold type. Notably, some metabolic ratios, such as Gln/tNAA, showed a statistically significant difference between TU and PT (e.g., *p* < 0.001 for Gln/tNAA), but the respective T1 and T2 values for MRF were not statistically significant. For a visualization of these data, see also Figure 3 and Figure 4.

Median Values in Different Regions of Interest					
Segmentations	TU	PT	TU vs. PT	TU + PT	NAWM
Quantity	Median (Q1, Q3)	Median (Q1, Q3)	*p*-Values	Median (Q1, Q3)	Median (Q1, Q3)
T1	1724 (1690, 1804)	1756 (1661, 1810)	0.773	1770 (1712, 1792)	950 (941, 972)
T2	85.5 (80.1, 105.8)	102.0 (90.0, 117.3)	0.272	101.6 (94.0, 106.0)	42.9 (42.6, 43.3)
tCho/tNAA	0.48 (0.42, 0.55)	0.38 (0.34, 0.44)	0.004	0.40 (0.39, 0.49)	0.20 (0.18, 0.21)
Gln/tNAA	0.61 (0.56, 0.70)	0.38 (0.35, 0.52)	0.001	0.43 (0.40, 0.50)	0.16 (0.13, 0.20)
Gly/tNAA	0.28 (0.20, 0.36)	0.20 (0.16, 0.24)	0.003	0.22 (0.18, 0.26)	0.07 (0.06, 0.10)
Ins/tNAA	1.15 (1.04, 1.21)	1.06 (0.90, 1.13)	0.030	1.09 (0.94, 1.14)	0.54 (0.51, 0.59)
tCho/tCr	0.69 (0.63, 0.80)	0.76 (0.66, 0.83)	0.867	0.72 (0.65, 0.81)	0.37 (0.35, 0.40)
Gln/tCr	0.90 (0.75, 1.31)	0.67 (0.58, 1.12)	0.042	0.70 (0.61, 1.31)	0.32 (0.26, 0.45)
Gly/tCr	0.51 (0.33, 0.64)	0.42 (0.28, 0.52)	0.024	0.42 (0.30, 0.55)	0.15 (0.10, 0.22)
Ins/tCr	1.98 (1.88, 2.53)	1.97 (1.85, 2.34)	0.471	1.95 (1.85, 2.21)	1.05 (0.96, 1.16)

## Data Availability

The datasets used during this study can be made available by the corresponding author upon reasonable request.

## Supplementary Materials

The following supporting information can be downloaded at: https://www.mdpi.com/article/10.3390/cancers16050943/s1, Figure S1: Sørensen-Dice Similarity Coefficients (DSCs) in the tumor segmentation between the segmentation (TU), the MRF’s relaxation time hotspots (T1, T2), and MRSI’s metabolite ratios (tCho/tNAA, Gln/tNAA, Gly/tNAA, Ins/tNAA). Gln/tNAA had the highest correspondence with the tumor segmentation and T1 and T2 hotspots; Figure S2: A: Metabolite ratios tCho/tNAA, Gln/tNAA, Gly/tNAA, and Ins/tNAA, as well as tCho/tCr, Gln/tCr, Gly/tCr, and Ins/tCr, for a hotspot threshold of 1.5, plotted separately for the tumor segmentation (TU), the tumor region and the peritumoral region (TU+PT), the peritumoral region alone (PT), and the normal-appearing white matter control region (NAWM). B: The same metabolite ratios for different hotspot thresholds, with one line each for TU, TU+PT, and PT; Figure S3: Overview of the volumes of the region of interest (ROI), the MRF hotspots (T1, T2), and the MRSI hotspots (tCho/tNAA, Gln/tNAA, Gly/tNAA, Ins/tNAA), in the tumor (A) and the peritumoral region (B); Figure S4: Example spectra of patient 10 (anaplastic astrocytoma, grade 3, female, 28 years of age). Normal appearing white matter spectrum (left) and tumor spectrum (right). Below, glutamine (Gln) maps overlaid with a T1w reference image are shown, and the voxel position is indicated; Table S1: Cohort overlap with previous publications [6,7]; Table S2: An overview according to the minimum reporting standards in MR spectroscopy [25]; Table S3: MRF sequence parameters.

## Abbreviations

AD	Acquisition delay
CE	Concentration estimates
Cho	Choline
COID	Center of intensity differences
Cr	Creatine
CRT	Concentric ring trajectories
DSC	Sørensen–Dice similarity coefficient
FID	Free induction decay
FISP	Fast imaging with steady-state precession
FLAIR	Fluid-attenuated inversion recovery
FOV	Field of view
Gln	Glutamine
Glu	Glutamate
Gly	Glycine
Ins	Myo-inositol
MP2RAGE	Magnetization-prepared 2 rapid gradient-echo
MRF	Magnetic resonance fingerprinting
MRI	Magnetic resonance imaging
MRSI	Magnetic resonance spectroscopic imaging
NAA	N-acetylaspartate
NAWM	Normal-appearing white matter
PET	Positron emission tomography
PT	Peritumoral segmentation
ROI	Region of interest
SNR	Signal to noise ratio
TA	Acquisition time
tCho	Total choline
tCr	Total creatine
TE	Echo time
tNAA	Total N-acetylaspartate
TR	Repetition time
TU	Tumor segmentation
