# Supplementary material for: A Comparison of 7 Tesla MR Spectroscopic Imaging and 3 Tesla MR Fingerprinting for Tumor Localization in Glioma Patients
Source: Cancers (Basel). 2024 Feb 26;16(5):943. doi: 10.3390/cancers16050943 (PMC10931074; doi:10.3390/cancers16050943)
Supplement: Supplementary file 1 [file cancers-16-00943-s001.zip › cancers-2750283-supplementary.pdf]

**Table S1.** Cohort overlap with previous publications [6,7].

| Cohort Overlap | | |
|---|---|---|
| Patient ID | | |
| This Study | Hangel 2020 | Hangel 2022 |
| 1 | 3 | - |
| 2 | 4 | 2 |
| 3 | 5 | 3 |
| 4 | 6 | 4 |
| 5 | - | 5 |
| 6 | 7 | - |
| 7 | - | 6 |
| 8 | 12 | 8 |
| 9 | 17 | - |
| 10 | 20 | 13 |
| 11 | - | 15 |
| 12 | - | 16 |

**Table S2.** An overview according to the minimum reporting standards in MR spectroscopy [25].

| Minimum Reporting Standards in MR Spectroscopy - Overview | |
|---|---|
| **Site** | Vienna High Field MR Center |
| **1. Hardware** | |
| **a. Field strength** | 7T |
| **b. Manufacturer** | Siemens |
| **c. Model** | Magnetom |
| **d. RF coils: nuclei (transmit/ receive), number of channels, type, body part** | 1H, 32 ch, head, Nova Medical |
| **e. Additional hardware** | N/A |
| **2. Acquisition** | |
| **a. Pulse sequence** | FID-MRSI |
| **b. Volume of interest (VOI) locations** | Tumor, peritumoral, NAWM |
| **c. Nominal VOI size** | 220×220×110 mm³ |
| **d. Repetition time (TR), echo time (TE)** | 450 ms / 1.3 ms acquisition delay |
| **e. Total number of excitations or acquisitions per spectrum** | N/A, spatial-spectral encoding |
| **In-time series for kinetic studies** | N/A |
| **i. Number of averaged spectra (NA) per time-point** | N/A |
| **ii. Averaging method (e.g., block-wise or moving average)** | N/A |
| **iii. Total number of spectra (acquired / in-time series)** | N/A |
| **f. Additional sequence parameters (spectral width in Hz, number of spectral points, frequency offsets); If STEAM: Mixing Time TM; If MRSI: 2D or 3D, FOV in all directions, matrix size, acceleration factors, sampling method** | BW 2778 Hz, 1920 spectral points, MRSI: 3D, 220×220×133 mm³, 64×64×39, spatial-spectral encoding |
| **g. Water suppression method** | WET |
| **h. Shimming method, reference peak, and thresholds for "acceptance of shim" chosen** | Standard shim + manual adjustment, water peak < 50 Hz |
| **i. Triggering or motion correction method** | N/A |
| **3. Data analysis methods and outputs** | |
| **a. Analysis software** | LCModel 6.3-1 |
| **b. Processing steps deviating from quoted reference or product** | N/A |
| **c. Output measure** | Ratio |
| **d. Quantification references and assumptions, fitting model assumptions** | Simulated in NMRScope-B, macromolecular background |
| **4. Data Quality** | |
| **a. Reported variables (SNR, linewidth (with ref.** | SNR and linewidths not reported |

| | |
|---|---|
| **peaks))** | |
| **b. Data exclusion criteria** | tCr SNR <5; tCr FWHM >0.15 ppm; metabolite Cramér-Rao lower bounds (CRLB) >40 % |
| **c. Quality measures of post processing model fitting** | CRLB |
| **d. Sample spectrum** | See Supp. Fig. 4 |

**Table S3.** MRF sequence parameters.

| **MRF Sequence Parameters** | |
|---|---|
| **Voxel Dimensions** | $1.0 \times 1.0$ mm² |
| **Matrix Size** | $256 \times 256$ |
| **Field of View** | $256 \times 256$ mm² |
| **Number of Slices** | 10–13 |
| **Slice Thickness** | 5 mm |
| **TE** | 2 ms |
| **TI** | 21 ms |
| **TR** | 12.14–15.00 ms |
| **TA** | 3:51–4:51 (min:sec) |
| **Acceleration Factor** | 24 (inner k-space) |
| | 48 (outer k-space) |
| **RX Bandwidth** | 400 kHz |
| **FA** | 0–74° |
| **Fat Saturation** | no |

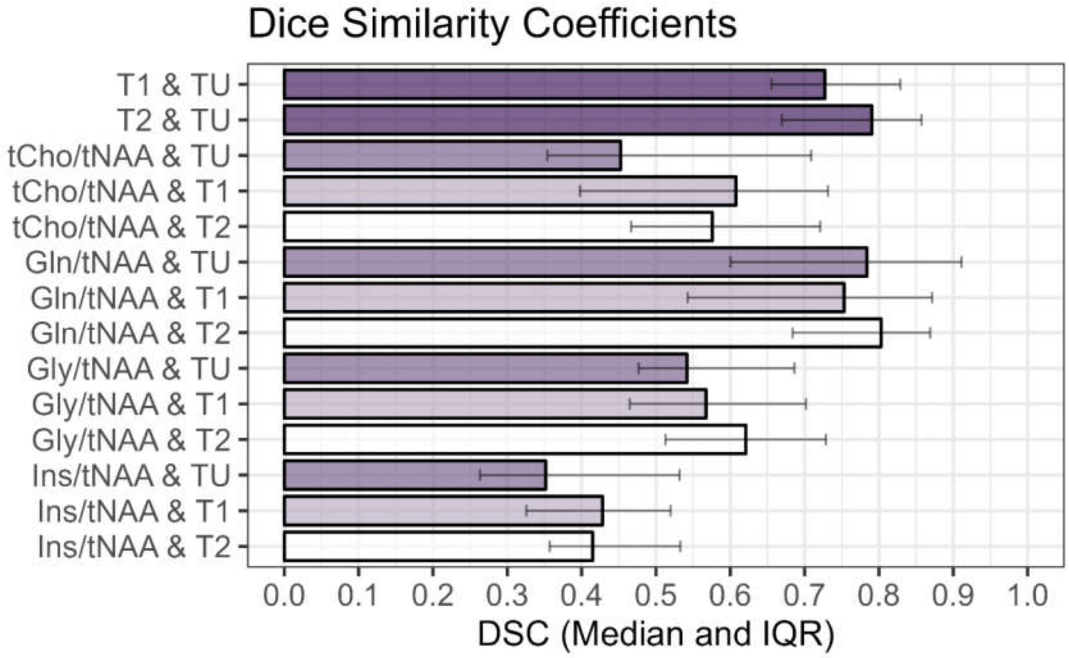


**Figure S1.** Sørensen-Dice Similarity Coefficients (DSCs) in the tumor segmentation between the segmentation (TU), the MRF's relaxation time hotspots (T1, T2), and MRSI's metabolite ratios (tCho/tNAA, Gln/tNAA, Gly/tNAA, Ins/tNAA). Gln/tNAA had the highest correspondence with the tumor segmentation and T1 and T2 hotspots.

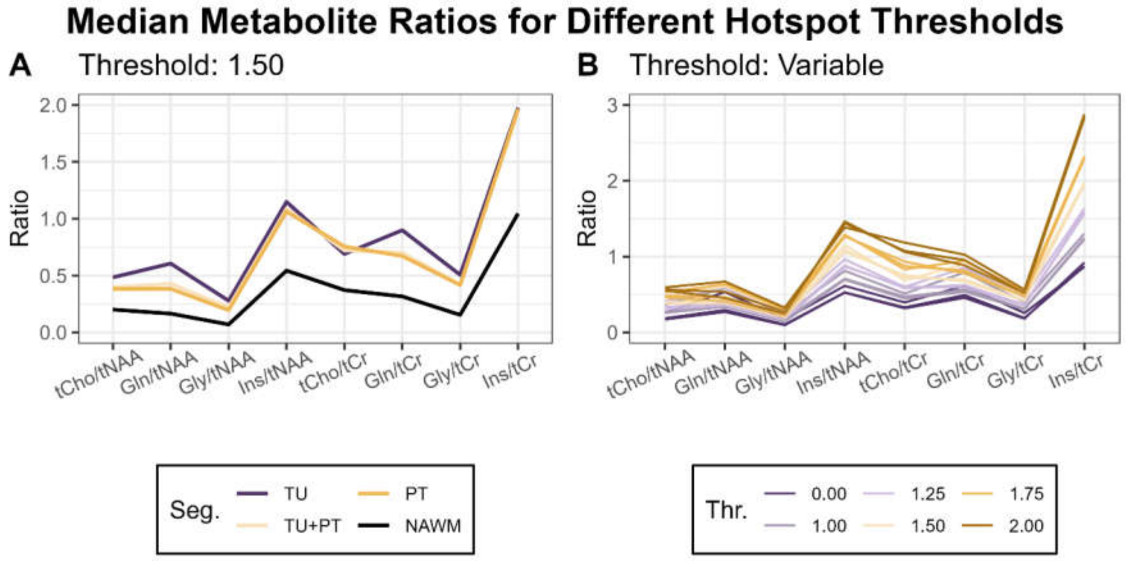


**Figure S2. A**: Metabolite ratios tCho/tNAA, Gln/tNAA, Gly/tNAA, and Ins/tNAA, as well as tCho/tCr, Gln/tCr, Gly/tCr, and Ins/tCr, for a hotspot threshold of 1.5, plotted separately for the tumor segmentation (TU), the tumor region and the peritumoral region (TU+PT), the peritumoral region alone (PT), and the normal-appearing white matter control region

(NAWM). **B**: The same metabolite ratios for different hotspot thresholds, with one line each for TU, TU+PT, and PT.

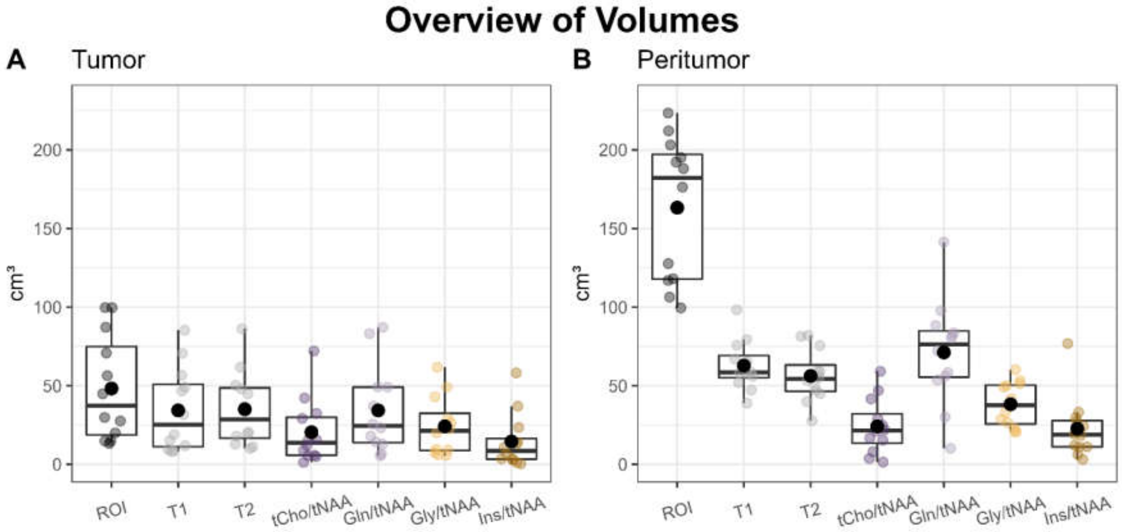


**Figure S3.** Overview of the volumes of the region of interest (ROI), the MRF hotspots (T1, T2), and the MRSI hotspots (tCho/tNAA, Gln/tNAA, Gly/tNAA, Ins/tNAA), in the tumor (**A**) and the peritumoral region (**B**).

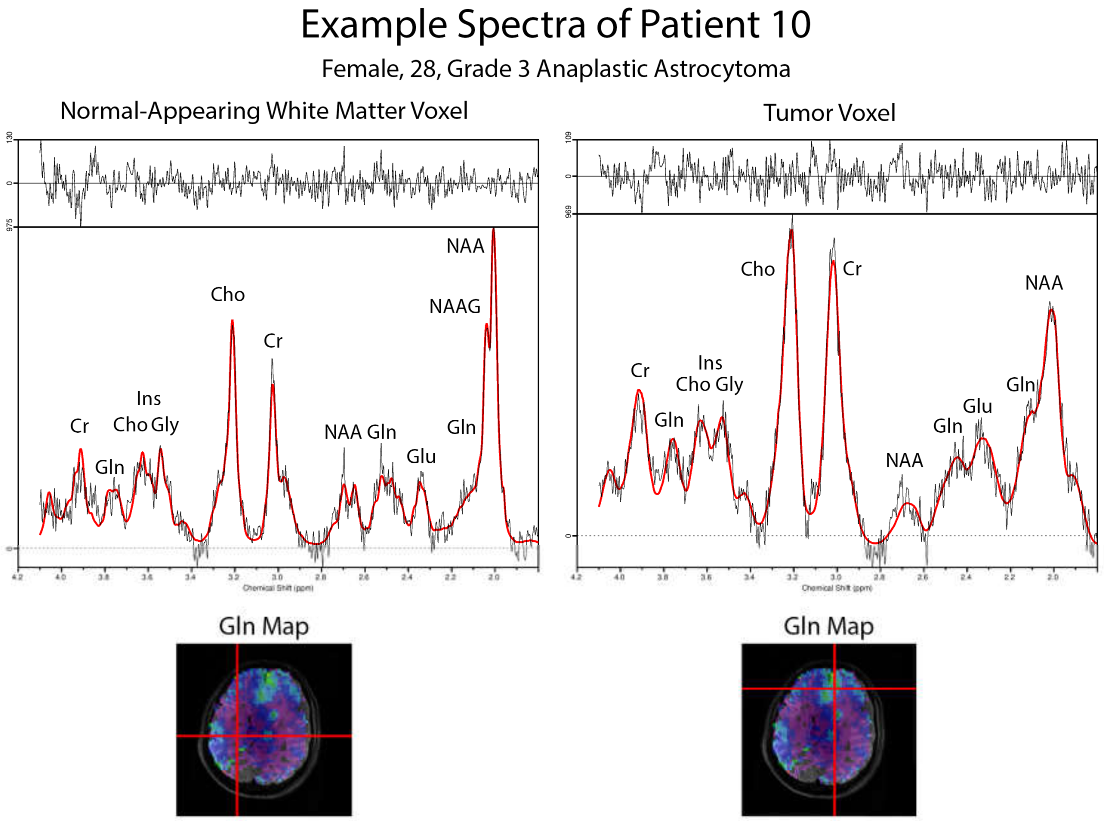


**Figure S4.** Example spectra of patient 10 (anaplastic astrocytoma, grade 3, female, 28 years of age). Normal appearing white matter spectrum (left) and tumor spectrum (right). Below, glutamine (Gln) maps overlaid with a T1w reference image are shown, and the voxel position is indicated.
